# Enhancing Visual Quality: The Impact of Alcohol-Assisted Delamination on Corneal Aberrations in Patients with Central Epithelial Basement Membrane Dystrophy

**DOI:** 10.3390/jcm14072342

**Published:** 2025-03-28

**Authors:** Marco Messina, Giuseppe Giannaccare, Carlo Cagini, Paolo Fogagnolo, Maria Poddi, Tommaso Bonifazi, Giuseppe Mirabella, Giulia Coco, Francesco Della Lena

**Affiliations:** 1 Department of Medicine and Surgery, Ophthalmology Section, S. Maria della Misericordia Hospital, University of Perugia, 06132 Perugia, Italy; carlo.cagini@unipg.it (C.C.); maria-poddi@libero.it (M.P.); tommaso.bonifazi@gmail.com (T.B.); gmirabella96@gmail.com (G.M.); francescodellalena@tiscali.it (F.D.L.); 2 Department of Surgical Sciences, Eye Clinic, University of Cagliari, 09127 Cagliari, Italy; giuseppe.giannaccare@gmail.com; 3 Department of Health Science, University of Milan, 20146 Milan, Italy; fogagnolopaolo@googlemail.com; 4 Department of Clinical Sciences and Translational Medicine, University of Rome Tor Vergata, 00133 Rome, Italy; giulia.coco@uniroma2.it

**Keywords:** epithelial basement membrane dystrophy, astigmatism, corneal aberrations, alcohol delamination of the corneal epithelium

## Abstract

**Background/Objectives**: Epithelial basement membrane dystrophy (EBMD) is a common corneal dystrophy characterized by recurrent corneal erosions and visual impairments due to surface irregularities and opacities. This study aims to evaluate the effectiveness of alcohol-assisted delamination (ALD) of the corneal epithelium in patients with EBMD affecting the visual axis, who experience decreased vision quality due to higher-order aberrations (HOAs) and irregular astigmatism. **Methods**: Eleven eyes of nine patients (four males and five females) were treated with ALD, with a mean age of 51.3 ± 19.7 years. All patients underwent refraction, best-corrected visual acuity (BCVA) assessment, a comprehensive slit-lamp examination for EBMD pattern identification, anterior segment imaging with and without fluorescein, tear break-up time (BUT) testing, corneal topography, corneal aberrometry (Zernike coefficients ($Z_{n}^{m}$) were calculated for a 5.0 mm simulated pupil), and anterior segment optical coherence tomography preoperatively and at 1-day, 14-day, 1-month, 3-month, 6-month, and 12-month intervals. **Results**: All patients demonstrated improvements in BCVA and visual quality, ocular comfort, and BUT results. The mean root mean square (RMS) value of total corneal aberrations decreased from 1.72 ± 0.90 μm to 0.90 ± 0.62 μm, while the mean RMS value of HOAs reduced from 0.92 ± 0.48 μm to 0.53 ± 0.28 μm. Astigmatism and trefoil were the aberration components that exhibited the most significant reductions. **Conclusions**: Alcohol-assisted delamination of the corneal epithelium is a safe and effective treatment for central EBMD patients experiencing visual quality deterioration. Astigmatism and trefoil appear to be the primary aberrations contributing to visual disturbances in this patient population.

## 1. Introduction

Epithelial basement membrane dystrophy (EBMD), also known as Cogan’s microcystic epithelial dystrophy or map-dot-fingerprint dystrophy, is a common condition affecting the anterior portion of the cornea. Its prevalence in the general population is greater than 2%, and some studies suggest an autosomal dominant mode of inheritance. However, not all affected individuals report a family history, indicating that the condition may also occur sporadically [1].

The pathogenesis of EBMD appears to stem from an intrinsic dysfunction of the basal epithelial cells, resulting in the secretion of an abnormal basement membrane that extends into the epithelium. This is accompanied by the accumulation of fibrillogranular material both between Bowman’s layer and the basement membrane and within the epithelium. The corneal surface irregularities created may be completely asymptomatic and often misdiagnosed; however, they can sometimes lead to significant symptoms such as photophobia, discomfort, foreign body sensation, and pain due to recurrent corneal erosions [2,3]. When these irregularities are near the visual axis or extensive across the surface, they may quantitatively and qualitatively affect vision [4].

Symptoms such as glare, ghosting, blurriness, poor contrast, halos, and starbursts are commonly reported when the visual axis is involved [5]. These symptoms are likely not only due to the irregular astigmatism induced by the abnormal deposits but also to higher-order aberrations (HOAs)—small optical irregularities in the eye that can impact visual quality. Unlike lower-order aberrations, these errors cannot be fully corrected with standard spectacles or most contact lenses [6].

Management of EBMD depends on the symptoms and the location of the deposits. For asymptomatic cases, a “watch and wait” approach is recommended. Depending on the severity of symptoms, first-line treatments may include topical lubricants, hypertonic solutions, or bandage contact lenses. Surgical options, such as epithelial debridement, phototherapeutic keratectomy (PTK), and alcohol-assisted delamination (ALD) of the corneal epithelium, are also well-documented for treating EBMD that is resistant to conservative management [7]. ALD is a technique designed to achieve a relatively atraumatic removal of the corneal epithelium by creating a cleavage plane between the epithelium and Bowman’s membrane. Consequently, it is increasingly utilized for a variety of corneal diseases. Although the efficacy of ALD in treating recurrent epithelial erosions due to EBMD has been established [8], no studies have specifically addressed its management in patients with central EBMD who experience visual symptoms related to HOAs.

Our study aims to rigorously evaluate the effectiveness of ALD in patients with EBMD who experience significant visual impairment primarily attributed to HOAs and irregular astigmatism. The presence of these optical aberrations can severely compromise visual quality, leading to difficulties in daily activities and a diminished quality of life. By systematically assessing changes in best-corrected visual acuity (BCVA), ocular comfort, and various corneal aberrations—including total corneal aberrations, HOAs, and specific components such as astigmatism and trefoil—both preoperatively and at multiple follow-up intervals, we aim to provide comprehensive insights into the efficacy of ALD as a therapeutic intervention. Our primary objective is to establish ALD not only as a safe procedural option but also as an effective means of addressing the underlying pathophysiological changes associated with EBMD. We hypothesize that patients undergoing ALD will experience marked improvements in visual acuity and a reduction in discomfort, correlating with a significant decrease in corneal aberrations. These changes are anticipated to enhance the overall visual quality and stability for patients, thereby addressing a critical unmet need in the management of EBMD.

Moreover, this study will contribute valuable data to the growing body of literature surrounding surgical interventions for EBMD, particularly in the context of ALD. By elucidating the procedural outcomes and potential benefits, our findings may inform clinical practice guidelines and help refine the selection criteria for patients who would benefit most from this intervention. Ultimately, this research aims to enhance patient outcomes and quality of life, fostering a better understanding of how ALD can be integrated into the therapeutic arsenal for managing EBMD effectively. To our knowledge, this is the first paper to demonstrate the safety and effectiveness of ALD as a first-line treatment option for central EBMD in patients who report unsatisfactory visual quality.

## 2. Materials and Methods

This research was conducted in accordance with the ethical principles established in the Declaration of Helsinki (2008). Participants provided oral consent and did not receive any financial compensation. The audit population was identified retrospectively through a review of medical records, focusing on patients diagnosed with central EBMD who underwent ALD treatment between 2023 and 2024 at our tertiary referral cornea service at Santa Maria della Misericordia Hospital in Perugia, Italy.

This study included nine patients (11 eyes) diagnosed with EBMD who were treated with ALD at our institution. All participants presented with significant visual disturbances and ocular discomfort, which were attributed to corneal irregularities associated with the disease. Notably, none of the patients had a history of recurrent corneal erosions, which can often complicate the clinical picture in EBMD. The essential criteria for inclusion in this study were a confirmed diagnosis of EBMD affecting the visual axis and the presence of visual symptoms, including decreased visual acuity and ocular discomfort, directly related to the corneal condition.

Conversely, patients meeting any of the following criteria were excluded from the study: a history of previous ocular surgeries, which could interfere with the assessment of ALD outcomes; the presence of glaucoma or any other significant ocular pathology that could confound the results; a diagnosis of keratoconus or suspected keratoconus, as these conditions have distinct management protocols and may influence corneal topography; and amblyopic eyes, to ensure that visual outcomes were solely attributable to the effects of EBMD and the ALD procedure.

The diagnosis of EBMD was established through a comprehensive clinical examination. A slit-lamp examination was performed on all patients, during which characteristic findings of EBMD were assessed. These findings included the presence of subepithelial microcysts, map-like changes, dot formations, or fingerprint patterns observed in the corneal epithelium, and the diagnosis was confirmed based on the distinct morphological characteristics identified during the examination.

Prior to undergoing ALD, all patients underwent a thorough preoperative evaluation, which included measurement of BCVA to establish baseline visual function, corneal topography and aberrometry to quantify the degree of corneal irregularity and HOAs, anterior segment optical coherence tomography (AS-OCT) to visualize the corneal layers and assess the extent of epithelial involvement, and evaluation of tear break-up time (BUT) to assess ocular surface stability and tear film quality. The alcohol-assisted delamination procedure was performed under sterile conditions.

Postoperative care included the use of antibiotic and anti-inflammatory eye drops to promote healing and reduce the risk of infection. Patients were followed up at 1 day, 14 days, 1 month, 3 months, 6 months, and 12 months postoperatively.

During follow-up visits, assessments included BCVA, ocular comfort evaluation, corneal topography, and measurement of HOAs to monitor changes and improvements in visual quality and corneal surface integrity.

### 2.1. Clinical Assessment and Data Collection

Each patient included in the study underwent a comprehensive baseline preoperative assessment during which the following data were collected: age, sex, ethnicity, refraction, BCVA, break-up time test (BUT), pattern of the EBMD (map, dot, or fingerprint, based on the predominant pattern observed in each eye), corneal topography and aberrometry (using the Sirius+ tomograph and corneal topographer, CSO, Florence, Italy), and images of the anterior segment obtained through a full slit-lamp examination (Digital Slit Lamp SL-9900, CSO, Florence, Italy). Additionally, anterior segment optical coherence tomography scans (SPECTRALIS Anterior Segment Module, Heidelberg Engineering, Heidelberg, Germany) were performed on all patients to confirm that any irregularities were confined to above Bowman’s layer and to exclude any involvement of the anterior stroma.

Corneal topography and aberrometry were conducted using the Scheimpflug-Placido Sirius topographer. For each eye, at least three images with optimal centering, focus, and continuous Placido disk lines were captured. Zernike coefficients ($Z_{n}^{m}$) were calculated for a 5.0 mm simulated pupil.

The following measurements were collected: root mean square (RMS) total corneal aberrations, RMS higher-order aberrations (HOAs), RMS astigmatism, RMS trefoil, RMS coma, RMS spherical aberration, and the Strehl ratio.

Root mean square (RMS) is a unit commonly used to quantify wavefront aberrations. It is calculated as the square root of the arithmetic mean of the squares of the distances of all points of the wavefront, expressed in microns, from the reference plane. RMS provides an estimate of the mean deviation of the wavefront from the ideal plane, with a higher value indicating a more aberrated wavefront.

The point spread function (PSF) describes how a point source of light is represented in an optical system, evaluating the dispersion of light caused by a surface when aberrations are present. The Strehl ratio, derived from the PSF, is defined as the ratio of the peak intensity of the actual PSF of the system to the peak intensity of the PSF of an ideal system limited only by diffraction over the system’s aperture (i.e., the pupil). A ratio of 1 indicates a perfect system with no aberrations; the higher the Strehl ratio, the better the optical performance of the system.

### 2.2. Surgical Procedure

All procedures were performed by the same expert surgeon (MM), ensuring consistency and expertise throughout the surgical process. Under an operative microscope, a lid speculum was carefully inserted to maintain proper exposure of the eye, and topical anesthesia was applied using lidocaine hydrochloride (4%, Alfa Intes Industria Terapeutica Splendore S.R.L., Casoria, Italy) to ensure patient comfort during the procedure.

Following anesthesia, a 10 mm optical zone marker was positioned on the cornea to delineate the area of treatment accurately. To facilitate the delamination process, approximately 4 to 5 drops of 20% alcohol (ethyl alcohol 95% IV f 10mL injectable preparation) (S.A.L.F. S.p.A., Cenate Sotto, Italy) were carefully placed within the confines of the zone marker. This alcohol was allowed to remain in contact with the corneal epithelium for a duration of 30 s, effectively loosening the epithelial layer. After this period, the alcohol was removed using a spear-shaped cellulose sponge, ensuring that any residual alcohol was thoroughly washed away from the eye with a balanced salt solution to prevent any potential toxicity. Once the epithelium was sufficiently loosened, it was gently peeled off using either a dry swab or a crescent blade, minimizing trauma to the underlying stroma.

To protect the corneal surface during the healing process, a bandage contact lens (BCL) (Safe-Gel 7 days, Safilens S.R.L., Staranzano, Italy) was applied, providing a moist environment conducive to epithelial regeneration. Following the surgical procedure, all patients received the same standardized postoperative regimen to promote healing and prevent infection. This regimen included chloramphenicol 5 mg/mL eye drops (Flogocyn, Laboratoires Thea, Clermont-Ferrand Cedex, France), administered as one drop four times a day, and hydrocortisone 3.35 mg/mL eye drops (Sofacor, Laboratoires Thea, Clermont-Ferrand Cedex, France), administered as one drop once daily, for the duration that the BCL was in place. Both types of eye drops were preservative-free, reducing the risk of further irritation to the ocular surface.

After a period of 14 days, once the new corneal epithelium had regrown adequately, the bandage contact lens was removed. To monitor the outcomes and overall healing, all patients were evaluated at multiple postoperative intervals, specifically at 1 day, 14 days, 1 month, 3 months, 6 months, and 12 months.

These evaluations included a comprehensive assessment of refraction, best-corrected visual acuity (BCVA), tear break-up time (BUT) test, corneal topography, corneal aberrometry, and slit lamp imaging, allowing for a thorough understanding of the surgical outcomes and patient recovery.

## 3. Results

A total of 11 eyes from 9 patients underwent ALD for EBMD with involvement of the visual axis and associated visual disturbances. The patients had a mean age of 51.3 ± 19.7 years, comprising four males (44.4%) and five females (55.6%). All patients demonstrated excellent recovery with improvements in visual acuity and regularization of the ocular surface over the entire follow-up. Patients had various presentations of EBMD, primarily among Caucasian individuals. The prevalent patterns included fingerprint, map, dots, and cysts. Most patients showed enhanced BCVA postoperatively, with notable improvements, such as patient 4’s right eye, which improved from 6/18 to 6/6, indicating significant visual recovery. Changes in spherical and cylindrical values were observed, with reductions in astigmatism for several patients, contributing to better visual quality. Postoperative BUT values were largely greater than 10 s, indicating improved ocular surface stability and comfort.

Improvements in simulated keratometry (Sim-K) values were noted, particularly in patient 4, whose values changed from −1.26 ax 151° to −0.56 ax 102°, reflecting a more regular corneal surface. Overall, the data suggest that ALD effectively improves visual acuity, ocular comfort, and corneal topography in patients with EBMD. These results support ALD as a viable surgical option for managing visual disturbances linked to EBMD, particularly in reducing irregular astigmatism. All these results are summarized in Table 1.

**Figure 1:**
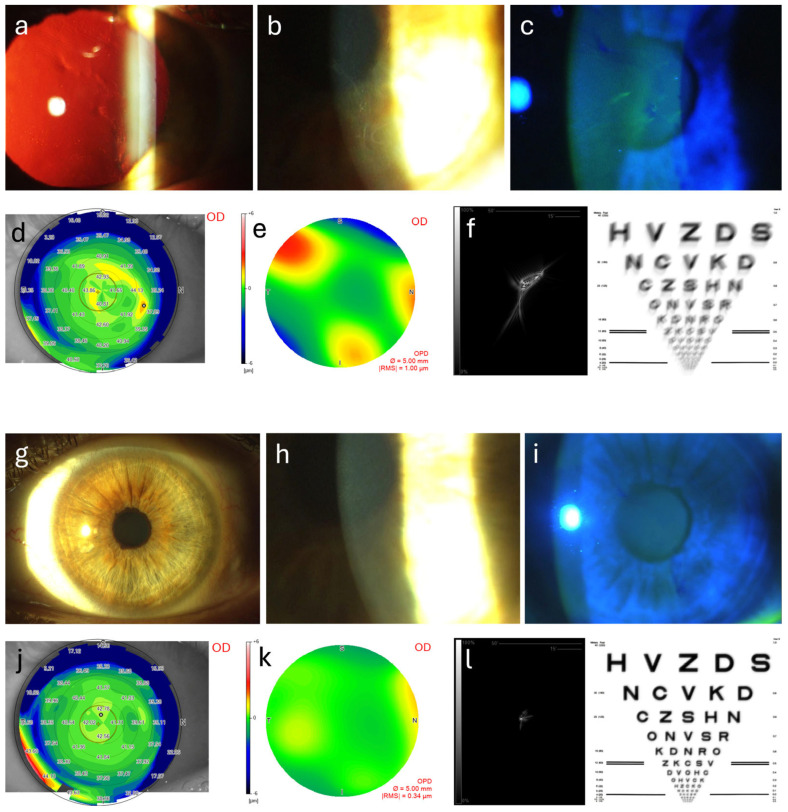
The figure compares preoperative (**a**–**f**) and postoperative (**g**–**l**) images of a patient with EBMD treated with ALD. Slit lamp images show preoperative visual axis involvement (**a**–**c**) and a complete clinical disappearance of corneal irregularities postoperatively (**g**–**i**). Topographic images illustrate the tangential anterior curvature with ocular surface irregularity before surgery (**d**) and its regularization afterward (**j**). Aberrometric optical path difference (OPD) maps depict changes in total corneal wavefront aberrations from the preoperative state (**e**) to 12 months post-surgery (**k**). Finally, the point spread function representation and visual acuity simulations demonstrate preoperative light dispersion and poor image quality (**f**), which significantly improve after ALD (**l**).

**Figure 2:**
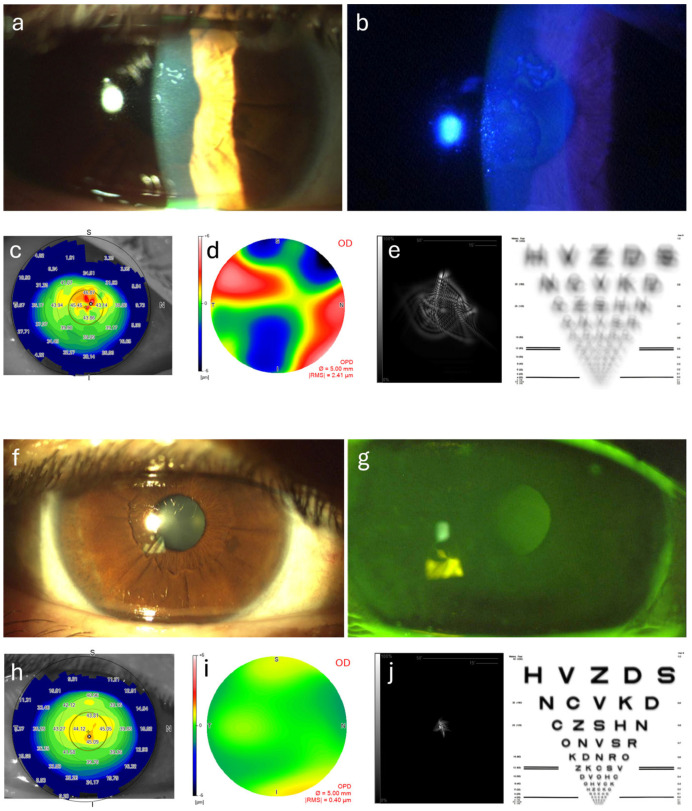
This figure illustrates another patient with EBMD before (**a**–**e**) and after (**f**–**j**) undergoing ALD. The preoperative slit lamp images clearly show central subepithelial involvement of EBMD (**a**,**b**), which completely resolves following the ALD procedure (**f**,**g**). Additionally, the preoperative tangential anterior curvature map (**c**) and total corneal aberrometric map (**d**) highlight the significant impact of subepithelial irregularities on the corneal surface and overall visual quality. The image quality reconstruction (**e**) further emphasizes these effects. Remarkably, at the 12-month follow-up, the postoperative images (**h**–**j**) demonstrate a considerable improvement in corneal surface regularity and visual clarity, showcasing the effectiveness of the treatment.

The data includes measurements of various optical aberrations (RMS Total, RMS HOAs, RMS astigmatism, RMS coma, RMS trefoil, and RMS spherical aberration) as well as the Strehl ratio, indicating the quality of the optical image produced by the eye. The findings show a significant reduction in total RMS aberrations from a preoperative mean of 1.72 ± 0.90 μm to 0.90 ± 0.62 μm postoperatively, suggesting a smoother corneal surface and improved optical quality. HOAs also decreased notably from a preoperative mean of 0.92 ± 0.48 μm to 0.53 ± 0.28 μm, indicating that ALD effectively reduces complex aberrations that can lead to visual disturbances. Additionally, RMS astigmatism showed a reduction from 1.39 ± 0.89 μm preoperatively to 0.67 ± 0.63 μm postoperatively, reflecting improved corneal regularity, which is crucial for enhancing visual acuity. The RMS coma values revealed a modest improvement, decreasing from 0.38 ± 0.23 μm to 0.33 ± 0.22 μm, while the RMS trefoil values decreased significantly from 0.42 ± 0.24 μm to 0.23 ± 0.17 μm, indicating enhanced optical quality. Spherical aberration also showed improvement, with preoperative mean values of 0.13 ± 0.12 μm reduced to 0.09 ± 0.09 μm postoperatively. The increase in the Strehl ratio from 0.0888 ± 0.0336 to 0.1782 ± 0.0685 indicates a substantial improvement in the quality of the optical image produced by the eye, correlating with enhanced visual outcomes. Overall, these findings demonstrate that ALD significantly reduces various optical aberrations and enhances the Strehl ratio in patients with EBMD, suggesting that ALD is an effective surgical intervention for improving both visual acuity and optical quality in this patient population. Aberrometric and topographic results are summarized in Table 2. Figure 1 and Figure 2 illustrate the changes in anterior segment appearance, the anterior corneal curvature topographic map, the total corneal aberration map, the point spread function, and the simulated visual acuity of two patients. As a demonstration that the significant reduction in the examined aberrations (Table 2) is a clear indicator of improved visual quality, all patients expressed general satisfaction with their postoperative visual condition.

## 4. Discussion

The aim of this study was to evaluate the efficacy and safety of ALD in reducing visual symptoms and aberrations associated with EBMD. ALD has been widely proven to be beneficial across various conditions, including recurrent corneal erosions (RCE), ocular surface neoplasias, early-stage Acanthamoeba keratitis, photorefractive keratectomy (PRK), corneal cross-linking (CXL), and Salzmann nodular degeneration (SND) [8,9]. The procedure has also shown benefits in certain types of corneal dystrophies, such as EBMD and granular dystrophy [10].

The application of ethanol facilitates the separation of the epithelium at the level of the epithelial basement membrane (EBM), creating a smooth surface underneath. The EBM consists of two distinct layers: the lamina lucida, which faces the epithelium, and the lamina densa, which is attached to the underlying Bowman’s layer [11]. The plane of separation induced by ethanol is likely located within the lamina lucida of the EBM, cleaving the hemidesmosomal junctions that connect the epithelial basal cells to the collagen VII anchoring fibrils [12]. This method allows for the creation of a uniform, loosely adherent epithelial sheet that can be removed with relative ease, while minimizing trauma to the lamina densa and Bowman’s layer.

While higher concentrations of alcohol can provoke inflammation and lead to keratocyte loss [13], the current standard procedure (30 s exposure to a 20% ethanol solution) has proven to be safe and effective for loosening the epithelium without causing additional corneal damage [12,13]. Mechanical epithelial debridement is a simple and cost-effective alternative; however, it has been shown to produce an irregular stromal surface, potentially disrupting Bowman’s membrane [14,15]. This can result in irregular epithelial healing and complications related to Bowman’s membrane irregularities, such as Salzmann nodular degeneration [16].

Damage to Bowman’s layer can also disrupt the corneal sub-basal plexus [17]. In fact, corneal nerves enter the stroma at the limbus and branch out toward the anterior stroma, organizing into a loose network at Bowman’s membrane level. Small branches perforate this layer to form the sub-basal nerve plexus, which extends to the superficial epithelial cells [18,19]. Preserving the integrity of Bowman’s membrane can prevent damage to the sub-basal plexus, thereby reducing postoperative pain and discomfort and shortening recovery time. Compared to manual mechanical debridement, ALD provides a smoother surface without disrupting Bowman’s layer, with minimal downsides in terms of time and cost.

Phototherapeutic keratectomy (PTK) is another alternative for corneal surface regularization in cases of EBMD and has been shown to be comparable to ALD in creating a smooth surface for epithelial regrowth [20]. However, PTK has several disadvantages compared to ALD, both economically and clinically. PTK alters the anterior corneal surface by ablating Bowman’s layer and a thin layer of stroma to remove irregularities, which can lead to corneal plexus disruption and longer recovery times. Limited anterior stromal ablation can also lead to an unwanted inflammatory and fibrogenic response, resulting in subepithelial corneal haze and potential visual complaints, as well as a hyperopic shift due to central corneal flattening if the ablation is confined to the central area [20,21]. Furthermore, PTK is more costly and typically performed only in specialized referral centers, whereas ALD is technically easier and more economically viable, allowing for safe execution in virtually any hospital without the need for subspecialized operators. Certainly, ALD has shown advantages over PTK [22], although the latter, according to recent studies and in combination with other surgical techniques, has still demonstrated some effectiveness [23].

EBMD, particularly when it affects the visual axis, can lead to aberrations that cause symptomatic visual disturbances. A study by Bellucci et al. [24] found that pseudophakic patients with EBMD had significantly lower BCVA and worse aberrometric indices compared to those without EBMD. Aldave et al. [25] demonstrated the effectiveness of mechanical epithelial debridement in alleviating visual symptoms in EBMD patients.

Aberrometers are used in ophthalmology to measure and analyze wavefront aberrations of the eye, assessing how light is distorted as it passes through the eye’s optical system. These devices project a light wavefront into the eye and analyze the reflected wavefront, revealing specific aberrations present in the eye. Corneal topographers and tomographers can conduct aberrometric analyses by simulating wavefront assessments based on topographic elevation data. While these systems are less precise because they cannot assess total aberrations originating from other ocular structures or evaluate certain aberrations like defocus and tilt, they have the advantage of being unaffected by pupil diameter or accommodation, which enhances the repeatability of measurements compared to conventional aberrometry. The cornea accounts for 70% of the total refractive power of the eye, and approximately 80% of all ocular aberrations originate from the cornea [26].

Since EBMD affects only the anterior surface of the cornea without impacting the inner surface or other ocular structures, its influence on wavefront aberrations can be reliably studied using a corneal aberrometer, as demonstrated in our case. Irregularities present in the anterior corneal surface, particularly those observed in conditions such as EBMD, can significantly affect the wavefront that passes through the cornea. These changes can lead to the induction of various optical aberrations, which may subsequently limit both the sharpness and contrast of the resulting visual images.

When these subepithelial anomalies occur along the visual axis, they can give rise to irregular astigmatism. This is primarily due to asymmetric alterations in the curvature of the anterior corneal surface, which disrupts the uniformity necessary for clear vision. As a result, the process of regularizing the anterior surface through techniques such as ALD can be beneficial. This procedure has the potential to reduce the irregular components of astigmatism, thereby restoring a more regular pattern of underlying astigmatism. Understanding this phenomenon provides valuable insight into the postoperative changes observed in corneal ΔK values and their associated axes. Such changes are critical for assessing the effectiveness of surgical interventions aimed at improving corneal topography and overall visual acuity.

Our study provides compelling evidence for the efficacy and safety of ALD in treating EBMD affecting the visual axis. The significant improvements observed in visual acuity and ocular surface regularization highlight the effectiveness of ALD in addressing the clinical challenges posed by this corneal condition.

Furthermore, the marked reduction in subjective symptoms, such as discomfort and visual disturbances, alongside enhanced tear break-up time, underscores the positive impact of ALD on patient outcomes and overall quality of life. The detailed analysis of corneal aberrations revealed substantial reductions in root mean square (RMS) values for total aberrations, HOAs, astigmatism, and trefoil.

These improvements were accompanied by a notable enhancement in the Strehl ratio, which is a critical measure of optical quality. Such changes are indicative of enhanced visual quality and clarity, which aligns with the high levels of patient satisfaction reported postoperatively. Patients expressed their contentment with the surgical outcomes, reflecting the positive transformation in their visual experiences. Importantly, the absence of complications throughout the follow-up period further strengthens the case for ALD as a safe intervention.

The lack of recurrence at one year post-surgery provides additional reassurance regarding the long-term effectiveness of this procedure for managing EBMD. These findings collectively establish ALD as not only a safe but also an effective intervention for patients suffering from this condition. As a result, the study supports the adoption of ALD as a viable treatment option in clinical practice, particularly for those patients whose quality of vision is significantly impacted by EBMD.

The study demonstrated that ALD significantly improved visual acuity and reduced subjective symptoms, such as discomfort and visual disturbances, likely due to the treatment’s ability to smooth the corneal surface and enhance tear film stability, resulting in lower corneal aberrations and a higher Strehl ratio, while also ensuring safety with no complications or recurrence of symptoms one year post-surgery.

Without ALD, patients with EBMD may experience persistent visual disturbances, discomfort, and deteriorating visual acuity due to ongoing corneal irregularities, leading to higher levels of corneal aberrations and a lower Strehl ratio, which can significantly impact their quality of life and may result in complications or progressive symptoms over time.

Future research involving larger sample sizes and extended follow-up will be essential to further elucidate the long-term benefits and potential applicability of this surgical approach across broader patient populations. Such investigations could provide deeper insights into the durability of the outcomes and help refine the criteria for patient selection, ultimately enhancing the standard of care for individuals affected by EBMD.

## 5. Conclusions

ALD has demonstrated effectiveness and safety in reducing symptoms and RCE, establishing it as a valuable option in the management of this condition. However, to our knowledge, no prior studies have specifically investigated its impact on visual symptoms and overall quality of vision in patients with central EBMD.

This study is noteworthy as it represents the first effort to evaluate the efficacy of ALD in treating visual disturbances in patients suffering from EBMD. Furthermore, it is the first to assess the significance of various corneal-induced aberrations on vision quality within this specific population, thereby filling a critical gap in the existing literature. While the present study provides valuable insights, it is important to acknowledge certain limitations. These include a small patient sample size, which may affect the generalizability of the findings, as well as a retrospective design that inherently limits the ability to draw definitive causal conclusions.

Additionally, there is limited existing literature on visual aberrations specifically related to EBMD, making it challenging to contextualize our results within a broader framework of research. To build upon these findings, further research with a larger cohort is needed to better understand the impact of EBMD on corneal aberrations and to validate our findings regarding the efficacy of ALD in enhancing visual acuity and quality of vision in EBMD patients. Such studies could provide deeper insights into the mechanisms by which EBMD affects visual function and help establish clearer criteria for treatment outcomes.

## Figures and Tables

**Table 1:** Demographic and clinical data of study patients.

Patient	Sex	Ethnicity	Age	Eye	EBMD Prevalent Pattern	Refraction		BCVA		BUT		Topographic ΔK (Sim-K)	
						Pre	Post	Pre	Post	Pre	Post	Pre	Post
1	M	Caucasian	42	Right	Fingerprint	−2.25 sph −0.75 ax 160° cyl	−2.25 sph −0.50 ax 180° cyl	6/6	6/5	>10	>10	−1.36 ax 156°	−0.88 ax 3°
				Left	Fingerprint	−1.75 sph −1.00 ax 20° cyl	−2.00 sph −0.50 ax 0° cyl	6/6	6/5	>10	>10	−1.68 ax 14°	−1.08 ax 177°
2	F	Caucasian	50	Right	Fingerprint	+0.75 sph	+0.50 sph +0.25 ax 90° cyl	6/9	6/6	6	>10	−0.12 ax 57°	−0.52 ax 16°
3	F	Caucasian	33	Left	Map	+1.75 sph +0.50 ax 135° cyl	+1.50 sph +0.50 ax 120° cyl	6/6	6/6	8	>10	−0.64 ax 46°	−0.47 ax 29°
4	F	Caucasian	55	Right	Dots	−5.50 sph −1.75 ax 130° cyl	−5.50 sph −0.25 ax 110° cyl	6/18	6/6	7	>10	−1.26 ax 151°	−0.56 ax 102°
5	M	Caucasian	69	Left	Map	+1.25 sph +1.00 ax 100° cyl	+1.50 sph +0.25 ax 110° cyl	6/24	6/12	>10	>10	−0.75 ax 26°	−0.51 ax 22°
6	F	Caucasian	68	Right	Map	−0.75 sph −1.50 ax 80° cyl	−1.00 sph −0.25 ax 70° cyl	6/12	6/6	8	>10	−1.31 ax 79°	−0.36 ax 58°
7	F	Arab	22	Right	Cysts	−3.00 sph −2.50 ax 175° cyl	−3.25 sph −2.00 ax 170° cyl	6/6	6/5	>10	>10	−2.85 ax 178°	−2.41 ax 2°
8	M	Caucasian	84	Right	Map	+0.50 sph +1.25 ax 170° cyl	+0.50 sph +1.25 ax 170° cyl	6/18	6/12	5	8	−1.30 ax 164°	−1.34 ax 164°
9	M	Caucasian	39	Right	Map	−1.00 sph −3.25 ax 0° cyl	−1.50 sph −2.50 ax 160° cyl	6/6	6/5	8	>10	−4.32 ax 174°	−3.60 ax 156°
				Left	Map	−1.50 sph −2.00 ax 10° cyl	−1.50 sph −1.25 ax 10° cyl	6/6	6/5	7	>10	−3.26 ax 12°	−1.91 ax 4°

M: male; F: female; EBMD: epithelial basement membrane dystrophy; Pre: preoperative; Post: postoperative (12 months); BCVA: best-corrected visual acuity; BUT: break-up time.

**Table 2:** Root mean square values of total corneal aberrations, higher order aberrations, single component aberrations, and Strehl ratio, both preoperatively and postoperatively.

Eye	RMS Total (μm)		RMS HOAs (μm)		RMS Astigmatism (μm)		RMS Coma (μm)		RMS Trefoil (μm)		RMS Sph Ab (μm)		Strehl Ratio	
	Pre	Post	Pre	Post	Pre	Post	Pre	Post	Pre	Post	Pre	Post	Pre	Post
1	1.00	0.34	0.73	0.26	0.69	0.22	0.18	0.18	0.56	0.09	0.19	0.04	0.0965	0.3253
2	1.33	0.68	0.44	0.51	1.26	0.44	0.23	0.25	0.27	0.39	0.16	0.05	0.1173	0.1952
3	0.79	0.64	0.77	0.62	0.21	0.15	0.55	0.57	0.18	0.16	0.00	0.01	0.1121	0.1631
4	0.88	0.48	0.67	0.44	0.58	0.19	0.36	0.31	0.48	0.21	0.00	0.08	0.1038	0.2640
5	2.41	0.40	1.55	0.27	1.84	0.30	0.34	0.10	0.16	0.17	0.06	0.01	0.0509	0.1860
6	0.62	0.57	0.33	0.30	0.52	0.48	0.04	0.07	0.25	0.03	0.09	0.09	0.1474	0.1420
7	2.84	0.38	1.73	0.30	2.25	0.24	0.44	0.19	0.92	0.14	0.13	0.07	0.0255	0.2020
8	2.73	1.63	0.42	0.37	2.69	1.59	0.20	0.17	0.22	0.22	0.14	0.11	0.0668	0.1391
9	1.33	1.36	0.97	0.99	0.91	0.93	0.58	0.60	0.49	0.65	0.08	0.08	0.0960	0.0905
10	3.04	2.24	1.48	0.90	2.66	2.05	0.87	0.71	0.68	0.24	0.45	0.34	0.0877	0.1056
11	1.94	1.21	1.02	0.91	1.65	0.79	0.41	0.45	0.40	0.24	0.08	0.07	0.0731	0.1479
**Mean ± SD**	**1.72 ± 0.90**	**0.90 ± 0.62**	**0.92 ± 0.48**	**0.53 ± 0.28**	**1.39 ± 0.89**	**0.67 ± 0.63**	**0.38 ± 0.23**	**0.33 ± 0.22**	**0.42 ± 0.24**	**0.23 ± 0.17**	**0.13 ± 0.12**	**0.09 ± 0.09**	**0.0888 ± 0.0336**	**0.1782 ± 0.0685**

RMS: root mean square; HOAs: higher order aberrations; Sph Ab: spherical aberration; pre: preoperative; post: postoperative (12 months); SD: Standard Deviation.

## Data Availability

The raw data supporting the conclusions of this article will be made available by the authors on request.
